# A dominant-negative F-box deleted mutant of E3 ubiquitin ligase, β-TrCP1/FWD1, markedly reduces myeloma cell growth and survival in mice

**DOI:** 10.18632/oncotarget.4120

**Published:** 2015-05-12

**Authors:** Ramaswamy Sharma, Paul J. Williams, Anjana Gupta, Brandon McCluskey, Shylesh Bhaskaran, Steve Muñoz, Babatunde O. Oyajobi

**Affiliations:** ^1^ Department of Cellular and Structural Biology, University of Texas Health Science Center at San Antonio, San Antonio, TX, USA; ^2^ Current address: Oklahoma Medical Research Foundation, Oklahoma City, OK, USA; ^3^ Current address: Vanderbilt Center for Bone Biology, Vanderbilt University Medical Center, Nashville, TN, USA; ^4^ Cancer Therapy and Research Center, San Antonio, TX, USA

**Keywords:** FWD1/β-TrCP1, 5TGM1, myeloma, plasmacytoma, proteasome

## Abstract

Treatment of multiple myeloma with bortezomib can result in severe adverse effects, necessitating the development of targeted inhibitors of the proteasome. We show that stable expression of a dominant-negative F-box deleted (ΔF) mutant of the E3 ubiquitin ligase, SCF^β-TrCP/FWD1^, in murine 5TGM1 myeloma cells dramatically attenuated their skeletal engraftment and survival when inoculated into immunocompetent C57BL/KaLwRij mice. Similar results were obtained in immunodeficient *bg-nu-xid* mice, suggesting that the observed effects were independent of host recipient immune status. Bone marrow stroma offered no protection for 5TGM1-ΔF cells in cocultures treated with tumor necrosis factor (TNF), indicating a cell-autonomous anti-myeloma effect. Levels of p100, IκBα, Mcl-1, ATF4, total and cleaved caspase-3, and phospho-β-catenin were elevated in 5TGM1-ΔF cells whereas cIAP was down-regulated. TNF also activated caspase-3 and downregulated Bcl-2, correlating with the enhanced susceptibility of 5TGM1-ΔF cells to apoptosis. Treatment of 5TGM1 tumor-bearing mice with a β-TrCP1/FWD1 inhibitor, pyrrolidine dithiocarbamate (PDTC), significantly reduced tumor burden in bone. PDTC also increased levels of cleaved Mcl-1 and caspase-3 in U266 human myeloma cells, correlating with our murine data and validating the development of specific β-TrCP inhibitors as an alternative therapy to nonspecific proteasome inhibitors for myeloma patients.

## INTRODUCTION

The ubiquitin (Ub)-proteasome system is a ubiquitous, multi-catalytic, macromolecular complex responsible for the non-lysosomal targeted degradation of many proteins essential to intracellular processes, including cell cycle progression, stress response, transcriptional activation, signal transduction, apoptosis, and DNA repair [1]. Polyubiquitination of protein substrates for proteasomal degradation is a multi-step sequential process involving Ub activation by the E1 enzyme and its transfer to an internal lysine residue on the substrate by E2 Ub conjugases and E3 Ub ligases [2]. Because these processes are often deregulated in cancer cells, perturbations of the Ub-proteasome pathway may lead to their apoptosis. This finding has led to the clinical development of proteasome inhibitors as anticancer agents and U.S. Food and Drug Administration approval of several proteasome inhibitors for the treatment of multiple myeloma [3]. However, use of non-specific inhibitors of proteasomal function results in obstruction of normal protein turnover and is associated with several adverse effects, including peripheral neuropathy, thrombocytopenia, severe hepatitis, pulmonary fibrosis, and pulmonary vasculitis resulting in respiratory failure [4]. Also, acquired resistance to bortezomib therapy is increasing [5-7]. Therefore, more selective targeting of the Ub-proteasome system with fewer side effects is still required.

Of the several hundred E3 Ub ligases, the SCF (Skp1-Cullin1-F-box protein) E3 ligase has been well-characterized [8]. The F-box motif of SCF confers specificity to the ubiquitination process by serving as the substrate recognition/recruitment domain for the E3 ligase. Approximately 70 F-box proteins have been identified, with β-TrCP (β-transducin repeats-containing protein) being the most well-studied. Two paralogs of β-TrCP, β-TrCP1 and β-TrCP2, are known and these share approximately 86% identical F-boxes [9]. SCF^β-TrCP1^ is involved in the Ub-dependent degradation of several regulatory proteins including key cell cycle and apoptotic proteins such as NF-κB, IκBα, caspase-3, ATF-4 and β-catenin [10-15]. Importantly, deregulation of NF-κB, a key substrate of SCF^β-TrCP1^ has been implicated in myelomagenesis and MM tumor progression [16, 17].

**Figure 1:**
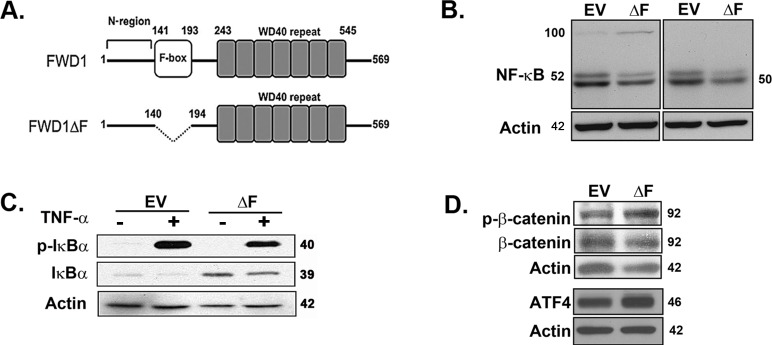
Dominant-negative expression of β-TrCP1/FWD1 disrupts NF-κB signaling in myeloma cells **A.** Representation of β-TrCP/FWD1ΔF (adapted from [12]). **B.** Immunoblotting for p50/105 (*left panel*) and p52/p100 (*right panel*) in lysates obtained from untreated 5TGM1-ΔF and 5TGM1-EV cells shows increased accumulation of p100 and decreased p52 levels in 5TGM1-ΔF myeloma cells. **C.** Cells were treated with 20 ng/ml TNF-α and lysates prepared. Immunoblotting shows increased basal levels of total IκBα in 5TGM1-ΔF cells. **D.** Untreated 5TGM1-ΔF and 5TGM1-EV cells were probed for ATF4, and for total and phosphorylated forms of β-catenin. Blots were normalized to actin.

To define the role of SCF^β-TrCP1^ specifically in multiple myeloma, we generated and characterized murine 5TGM1 multiple myeloma cells stably expressing a dominant negative mutant of the murine homolog of β-TrCP1 (FWD1; F-box/WD40-repeat protein) that lacks the F-box (FWD1ΔF; Figure 1A) [10, 12, 18]. We also used a small molecule synthetic inhibitor of β-TrCP1/FWD1 Ub ligase activity to validate our studies in human myeloma cells *in vitro* as well as *in vivo* in mice. Collectively, our data point to a critical role for β-TrCP1/FWD1 in myeloma growth and progression *in vivo*, thereby identifying a potential target for selective inhibition of the proteasome in patients with multiple myeloma. Because genetic inhibition of both β-TrCP paralogs in mice does not result in an overt phenotype beyond reversible testicular pathology [19], targeting β-TrCP in myeloma patients is unlikely to be associated with significant side effects and offers a viable alternative to general proteasome inhibitors.

## RESULTS

### Dominant-negative expression of β-TrCP1/FWD1 leads to accumulation of its substrates

We initially verified the effect of dominant-negative ΔF on known substrates of β-TrCP1/FWD1 in 5TGM1 myeloma cells. Immunoblotting for NF-κB subunits showed p100 accumulation and decreased p52 levels in 5TGM1-ΔF cells, consistent with a block in the β-TrCP1/FWD1-mediated processing of p100 to p52 (Figure 1B; panel 1). We could not detect p105 in either cell line, but p50 was also decreased in 5TGM1-ΔF cells (Figure 1B; panel 2). Also, total IκBα level was higher in the 5TGM1-ΔF cells than in 5TGM1-EV cells (Figure 1C), again consistent with a ΔF-mediated disruption in targeting of IκBα for Ub-mediated proteasomal degradation. Finally, we observed an increase in the accumulation of phosphorylated β-catenin and ATF4 (Figure 1D) that are known targets of β-TrCP1/FWD1 [12, 13]. Together, these results validate the inhibition of β-TrCP1/FWD1 in 5TGM1 cells.

### Tumor burden is significantly reduced in mice bearing 5TGM1-ΔF myeloma cells

**Figure 2:**
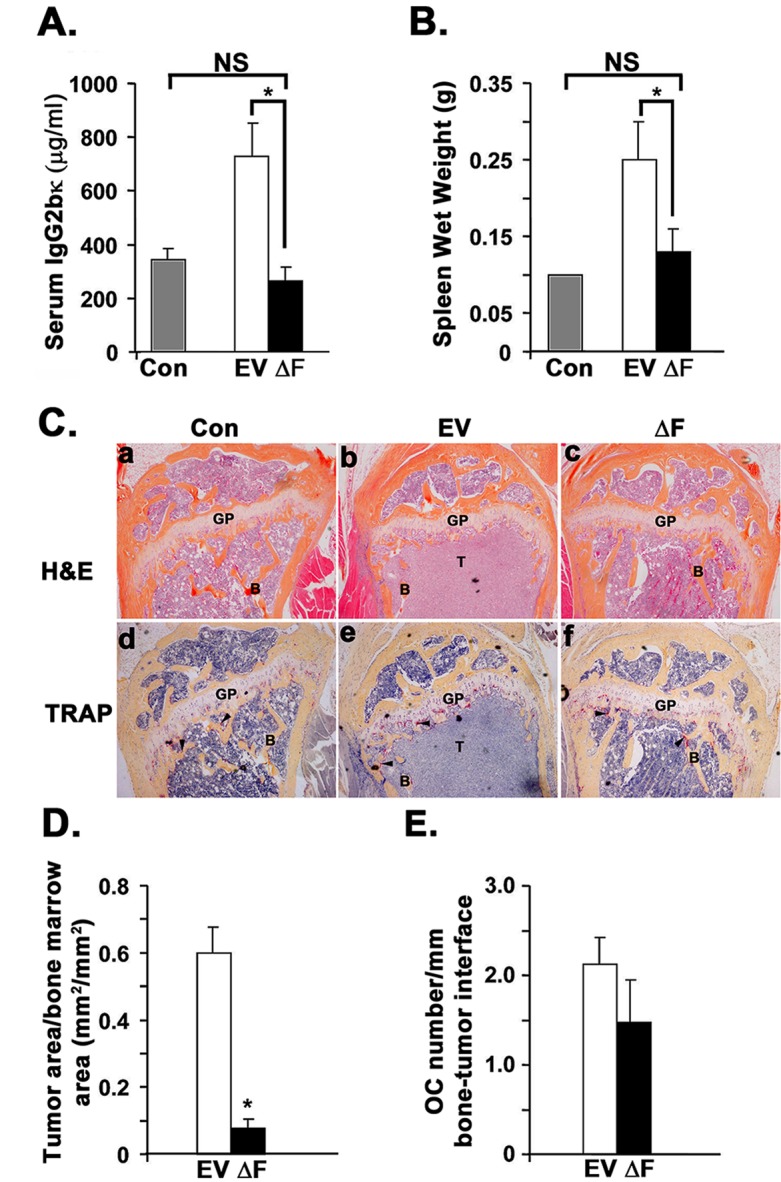
Tumor burden is significantly reduced in disseminated myeloma mouse model bearing 5TGM1-ΔF myeloma cells Control (Con) represents normal non-tumor-bearing mice injected with saline (n=4); EV = 5TGM1-EV-injected mice (*n* = 10); ΔF = 5TGM1-ΔF-injected mice (*n* = 10). **A.** 5TGM1 tumor burden assessed by serum IgG2bκ titer. **B.** Spleen wet weight at time of sacrifice. All mice had unequivocal evidence of myeloma tumor cells in spleen on hematoxylin and eosin (H&E)-stained sections. **C.** Representative photo-micrographs of serial sections of proximal tibial metaphyses from control mice (a,d) and mice intravenously inoculated with 5TGM1-EV (b,e) or 5TGM1-ΔF (c,f) myeloma cells stained either with H&E (a-c) or for tartrate-resistant acid phosphatase activity (TRAP; pinkish-red stain) to identify multi-nucleated osteoclasts (d-f). H&E-stained sections clearly show significantly increased tumor area in the bone marrow of mice inoculated with 5TGM1-EV mice **B.** as compared to control **A.** or 5TGM1-ΔF **C.** mice. GP= Growth Plate; B=trabecular bone; T=tumor. Arrowheads point to osteoclasts **D.** Tumor area per bone marrow area assessed by bone histomorphometry in the above H&E-stained sections of long bones (Counts of mice with no clearly discernible myeloma tumor in at least one leg bone: EV: 2/10; ΔF: 8/10. **E.** Osteoclast density represented as counts of tartrate-resistant acid phosphatase (TRAP^+^) multinucleated osteoclasts (OC; shown above) per mm bone tumor interface. In all cases, data represent mean ± SEM. NS, not significantly different; *, *P* < 0.05.

We next used a disseminated myeloma bone disease model to determine whether dominant-negative ΔF directly influences multiple myeloma cell growth and survival *in vivo*. Serum IgG2bκ titer, a marker of overall myeloma tumor burden, was significantly lower after 30 days in mice inoculated with 5TGM1-ΔF cells than in 5TGM1-EV-bearing mice (Figure 2A). This effect was not due to perturbed IgG2bκ production in 5TGM1-ΔF cells; we verified that monoclonal IgG2bκ levels were not different in conditioned media harvested from 5TGM1-EV and 5TGM1-ΔF cells before inoculation in mice (data not shown). Splenomegaly occurs in most disseminated mouse models of myeloma. We observed a significant reduction in splenic wet weights in 5TGM1-ΔF-bearing mice compared with 5TGM1-EV mice (Figure 2B). Furthermore, bone histomorphometric analysis revealed markedly reduced tumors in 5TGM1-ΔF mice (Figure 2C, panels a-c and Figure 2D). No discernible myeloma in either femur or tibia or in both leg bones were observed in 2 of 10 5TGM1-EV bearing mice as compared to 8 of 10 5TGM1-ΔF-bearing mice. Consistent with these findings of reduced skeletal tumor load, a trend toward reduced osteoclast numbers per tumor interface emerged in 5TGM1-ΔF mice (Figure 2C, panels d-f and Figure 2E), manifesting as fewer radiographically detectable lytic lesions (data not shown). Histomorphometry also indicated significantly reduced myeloma tumor infiltration in spleens of 5TGM1-ΔF mice. To exclude the possibility that host-initiated immune responses in immunocompetent C57BL/KaLwRij mice influenced engraftment of ΔF-expressing cells, we repeated the experiments in immunodeficient *bg-nu-xid* mice. We observed similar effects in (1) tumor in bone, (2) tumor in spleen, and (3) circulating monoclonal paraprotein titers (data not shown), suggesting that effects of the ΔF mutant on myeloma growth and survival *in vivo* are independent of perturbations in the immune compartment.

### ΔF mutant attenuates myeloma cell growth in a cell-autonomous manner

**Figure 3:**
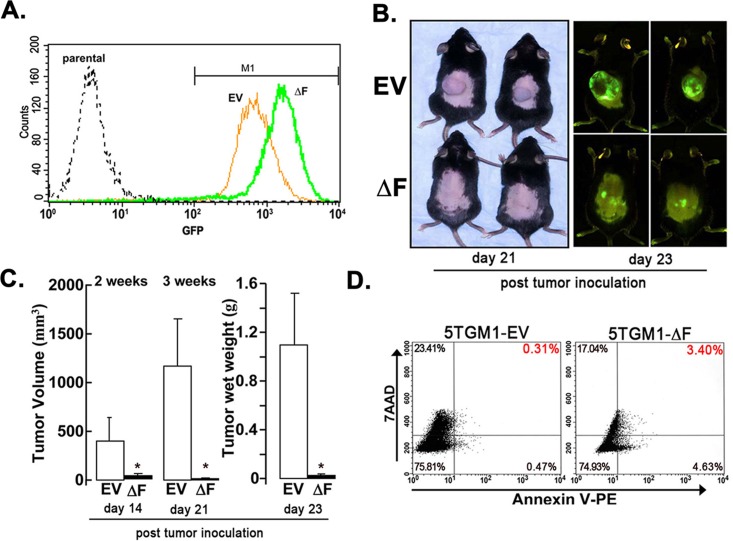
ΔF mutant attenuates myeloma cell growth in a cell-autonomous mode A subcutaneous plasmacytoma model, in which tumor cells were inoculated subcutaneously in flank of syngeneic naïve mice, was used to determine the role of the bone marrow microenvironment. **A.** GFP expression of 5TGM1-EV and 5TGM1-ΔF cells analyzed by flow cytometry immediately before inoculation in mice. A single peak for each cell type indicates relative homogeneity of GFP expression in either population. Consistent with this observation, median GFP expression of the gated M1 populations did not differ significantly from the median GFP for all cells. **B.** Mice were inoculated subcutaneously with 5TGM1-EV cells (EV, *n* = 5) or 5TGM1-ΔF cells (ΔF, *n* = 5). Representative mice are shown (*left panel*); whole-body optical images of tumor-emitting green fluorescence in anesthetized live mice inoculated with above cells taken post-tumor inoculation (*right panel*). Tumor growth in mice inoculated with 5TGM1-ΔF cells (*bottom*) was markedly inhibited compared with those inoculated with 5TGM1-EV cells (*top*). **C.** Quantitative analysis of tumor volume (days 14 and 21 post-tumor cell inoculation) and excised tumor wet weight (at the end of the experiment; day 23 post-tumor cell inoculation). Days 14, 21 and 23 refer to the number of days after tumor cell inoculation in the flank of mice. Expression of ΔF in 5TGM1 cells almost completely inhibited plasmacytoma growth *in vivo.* Data represent mean ± SEM; *, *P* < 0.05. **D.** Tumor tissue was harvested from 5TGM1-EV- and 5TGM1-ΔF-injected mice, disaggregated, sieved, stained with annexin V-phycoerythrin and 7-AAD, and analyzed by flow cytometry to quantify apoptotic cells (annexin V^+^, 7-AAD^−^; lower-right quadrant). Apoptotic cells in tumor tissue harvested from 5TGM1-ΔF-inoculated mice increased by 10-fold compared with 5TGM1-EV tumors.

The bone marrow microenvironment plays a critical role in myeloma cell growth and survival [20]. To determine whether the ΔF-induced attenuation of myeloma cell growth in bone *in vivo* was cell autonomous or due to tumor-induced changes in the bone marrow microenvironment, we used a subcutaneous, solitary plasmacytoma model in which tumor develops independent of marrow stroma. In this model, 5TGM1-EV tumors grew exponentially two weeks after tumor cell inoculation. By contrast, growth of 5TGM1-ΔF cells was almost completely inhibited, decreasing tumor volume and tumor wet weight (Figure 3B, 3C). This effect occurred despite inoculation of equal numbers of GFP-expressing cells (Figure 3A). Flow-cytometric analysis of harvested tumor cells revealed a 10-fold increase in apoptosis in 5TGM1-ΔF plasmacytomas compared with 5TGM1-EV tumors (Figure 3D). Overall, these data suggest that the profound antimyeloma effect of the dominant-negative FWD1ΔF is most likely independent of local signals emanating from the bone marrow microenvironment.

### BMSCs do not protect 5TGM1-ΔF myeloma cells from TNF-α-induced apoptosis

**Figure 4:**
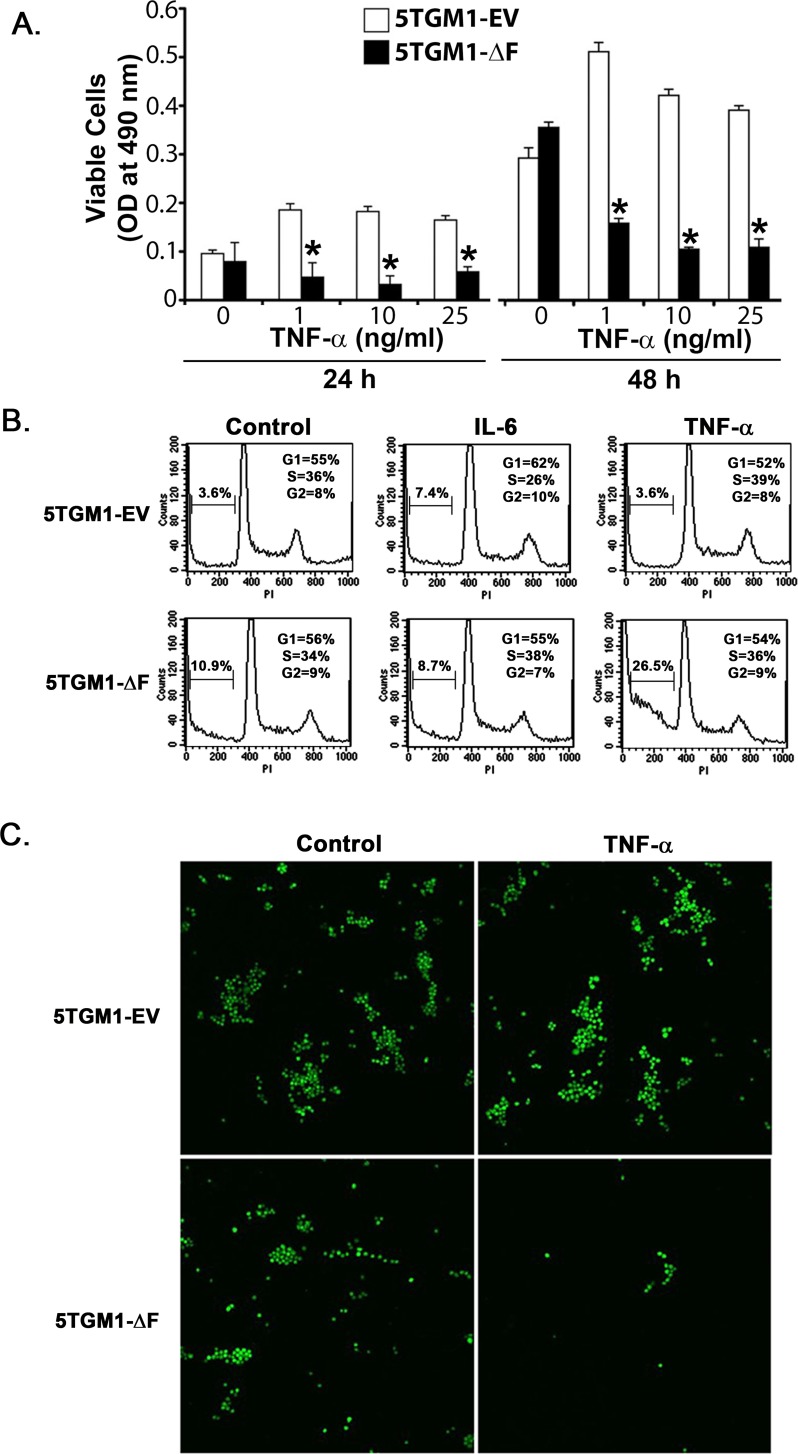
Bone marrow stromal cells do not protect 5TGM1-ΔF myeloma cells from TNF-α-induced apoptosis **A.** Cytotoxic and proliferative effects of TNF-α on 5TGM1-EV and 5TGM1-ΔF myeloma cells were evaluated in medium containing 2% FBS with a standard MTS assay. Data represent mean ± SEM; *, *P* < 0.05. **B.** 5TGM1-EV or 5TGM1-ΔF cells were treated with either 100 ng/ml of IL-6 or 20 ng/mL of TNF-α for 24 h, washed them in PBS, and analyzed for apoptosis by flow cytometry after staining with propidium iodide. **C.** 5TGM1-EV or 5TGM1-ΔF cells were grown on a layer of 14M1 BMSCs and visualized for GFP expression after treatment with 20 ng/ml of TNF-α for 72 h. Viability of 5TGM1-ΔF cells decreased irrespective of the presence of BMSCs.

To begin to explore the mechanism(s) underlying the cell-autonomous effect of the FWD1ΔF mutant, we focused on TNFs secreted by multiple myeloma cells [21]. Human and murine myeloma cells, including 5TGM1 cells, secrete both TNF-α and TNF-β [22-25], which can act as autocrine growth and survival factors [25, 26]. We reasoned that continuous TNF production by 5TGM1 cells as they grow *in vivo* may increase their levels locally, which would in turn induce apoptosis of 5TGM1-ΔF cells because of lack of NF-κB [27, 28]. Indeed, inhibition of NF-κB activity sensitizes B-lineage cells to the apoptosis-inducing effects of TNFs [29, 30]. This behavior would be consistent with the increased apoptotic cell death observed *in vivo* in 5TGM1-ΔF plasmacytomas in relation to the 5TGM1-EV plasmacytomas (Figure 3D). We therefore tested 5TGM1-EV and 5TGM1-ΔF cells with TNF-α for 24 to 48 h and used a standard MTS assay to assess cytotoxic effects. TNF-α decreased viability of 5TGM1-ΔF in a dose- and time-dependent fashion in relation to 5TGM1-EV cells (Figure 4A); we observed a similar pattern for TNF-β (data not shown). The apoptotic effect of low-dose TNF-α in 5TGM1-ΔF cells was more profound at lower cell density. Concentrations of TNF-α as low as 1 ng/ml induced cytotoxicity of low-density 5TGM1-ΔF cells but stimulated proliferation of 5TGM1-EV cells, confirming previous data [21]. As would be expected in cells with impaired NF-κB activity, 5TGM1-ΔF cells were at least 2 log orders more sensitive to high doses of both TNFs than were 5TGM1-EV cells. Cell cycle analyses indicated an approximate threefold increase in apoptosis in untreated ΔF cells compared with 5TGM1-EV cells (Figure 4B). Treatment with TNF-α further doubled apoptotic fraction compared with vehicle-treated ΔF cells and increased apoptosis approximately sevenfold when compared with TNF-α-treated 5TGM1-EV cells (Figure 4B). This effect appeared to be specific to TNF-α because treatment with recombinant murine interleukin 6 (IL-6) had little effect on apoptosis of 5TGM1-ΔF cells. Taken together, these data strongly suggest that TNFs may be involved in the inhibitory effect of FWD1 on myeloma survival.

In addition to supplying a tumor-permissive environment for myeloma growth, bone marrow cells play a critical role in protecting myeloma cells from apoptosis [31]. Therefore, we determined whether coculturing 5TGM1-ΔF cells with 14M1 BMSCs (isolated from a 5T mouse myeloma [20]) protected them from TNF-α-induced apoptosis. Imaging for GFP expression indicated fewer viable 5TGM1-ΔF cells than 5TGM1-EV cells in vehicle-treated cocultures. Even more striking was the effect of TNF-α; upon exposure to TNF-α, considerable cell death occurred in 5TGM1ΔF cells but not in 5TGM1-EV cells grown on 14M1 cell layers. This finding indicates that BMSCs do not protect ΔF cells from the apoptosis-inducing effect of TNF-α, as would be expected with deficient NF-κB activity (Figures 4C and S1).

### 5TGM1-ΔF myeloma cells exhibit constitutive upregulation of proapoptotic factors

**Figure 5:**
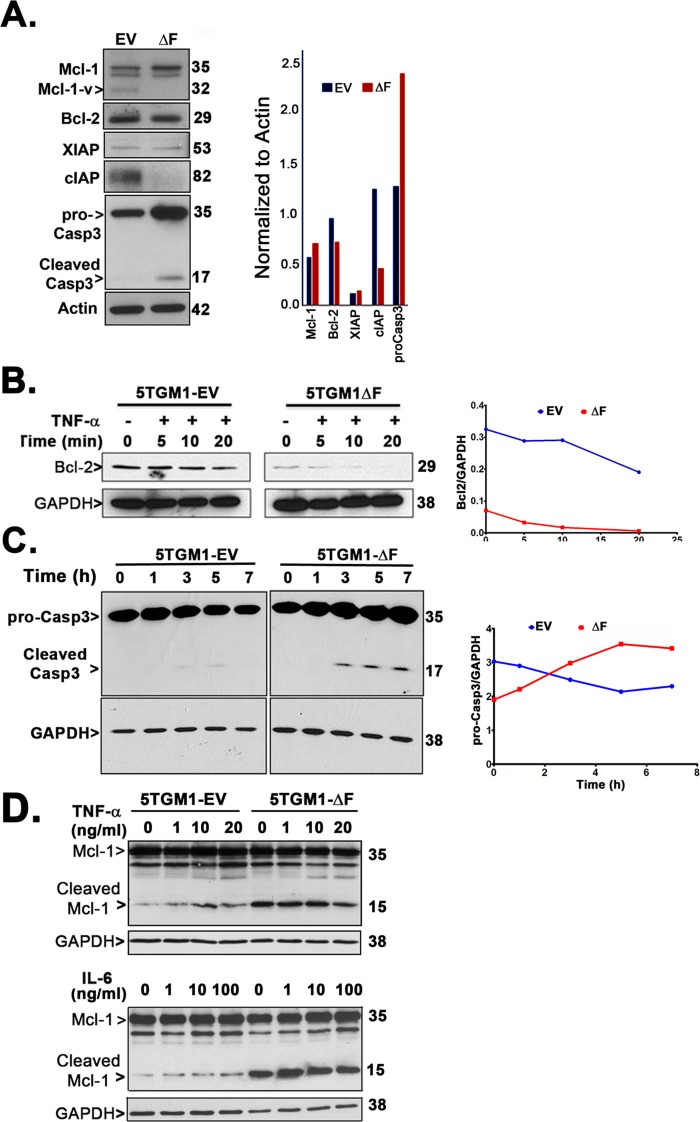
5TGM1-ΔF myeloma cells exhibit constitutive upregulation of proapoptotic factors **A.** Untreated 5TGM1 or 5TGM1-ΔF cells were probed for Mcl-2, Bcl-2, XIAP, cIAP and caspase-3 and normalized to actin. **B.** Western blotting of cell lysates shows reduced steady-state level of Bcl-2 protein in 5TGM1-ΔF cells that is rapidly cleared over time after treatment with 20 ng/ml TNF-α. **C.** Cells were treated with 10 μg/ml of cycloheximide for the indicated time points and harvested; immunoblotting of cell lysates shows significantly elevated half-life of procaspase-3 and time-dependent increase in cleaved caspase-3 in untreated 5TGM1-ΔF cells. **D.** Cells were treated for 15 min with TNF-α (0, 1, 10, and 20 ng/ml; *top panel*) or IL-6 (0, 1, 10, and 100 ng/ml; *bottom panel*) and harvested after 3 h into RIPA buffer followed by immunoblotting. Cleaved products of Mcl-1 are increased in untreated 5TGM1-ΔF cells.

Because we observed two-fold increase in TNF-α-mediated apoptosis of 5TGM1-ΔF cells *in vitro* (Figure 4B) and *in vivo* (Figure 3D), we determined the expression of known β-TrCP1/FWD1-regulated apoptotic proteins: Mcl-1 [32], Bcl-2 [33] and caspase-3 [15, 34-36]. We also analyzed the expression of anti-apoptotic proteins downstream of NF-κB, including XIAP and cIAPs [37, 38]. Mcl-1 is an antiapoptotic member of the Bcl-2 family that is essential for myeloma cell survival [39, 40]. We find increased levels of Mcl-1 in untreated ΔF cells (Figure 5A), consistent with other studies that show that proteasome inhibition can lead to Mcl-1 accumulation [41]. Interestingly, an isoform of Mcl-1, Mcl-1v, that lacks 46 amino acids in its N-terminal and exhibits higher anti-apoptotic potential as compared to Mcl-1 [42], appears to be downregulated in 5TGM1-ΔF cells. XIAP is highly expressed in myeloma cells [43] and is induced by IL-6 and IGF-1-mediated activation of NF-κB/MAPK/PI3K pathways [44]. Similarly, cIAPs also play an important role in NF-κB-mediated protection from TNF-α-induced apoptosis in myeloma cells [37, 45]. 5TGM1-ΔF cells did not exhibit changes in XIAP levels; however, cIAP was significantly reduced in untreated 5TGM1-ΔF cells than in 5TGM1-EV cells (Figure 5A). Bcl-2 undergoes TNF-α-mediated dephosphorylation and Ub-mediated proteasomal degradation [33]. Bcl-2 is also a potential transcriptional target of p100 and p52 [46]. As expected, Bcl-2 expression was lower in 5TGM1-ΔF cells (Figure 5A), consistent with a ΔF-induced disruption of the p100-p52 axis. Adding TNF-α further reduced Bcl-2 levels in 5TGM1-ΔF cells in comparison with 5TGM1-EV cells (Figure 5B), suggesting that TNF-induced reduction in Bcl-2 levels may also contribute to apoptosis of 5TGM1-ΔF cells *in vivo*. Next, we determined if pro-apoptotic caspase-3 expression differed between 5TGM1-EV and 5TGM1-ΔF cells. Higher levels of pro-caspase-3 as well as cleaved caspase-3 were observed in untreated ΔF cells. β-TrCP1 is known to reduce the half-life of procaspase-3 by promoting its ubiquitin-mediated proteasomal degradation [15]. Accordingly, time course experiments showed higher levels of procaspase-3 and cleaved caspase-3 in cycloheximide-treated 5TGM1-ΔF cells than in 5TGM1-EV cells (Figure 5C), indicating that decreased degradation lead to increased procaspase-3 accumulation in 5TGM1-ΔF cells. Active caspases can cleave full-length Mcl-1 [47, 48] to generate a proapoptotic fragment that induces Bax translocation to the mitochondria [49], thereby triggering apoptosis. Indeed, immunoblotting of untreated and TNF-α-treated or IL-6-treated 5TGM1-ΔF cell lysates showed constitutively higher levels of cleaved Mcl-1 (Figure 5D), thereby explaining their increased proclivity for apoptosis. Neither TNF-α nor IL-6 altered Mcl-1 levels significantly.

### Inhibition of β-TrCP by PDTC significantly reduces overall tumor burden in myeloma-bearing mice

**Figure 6:**
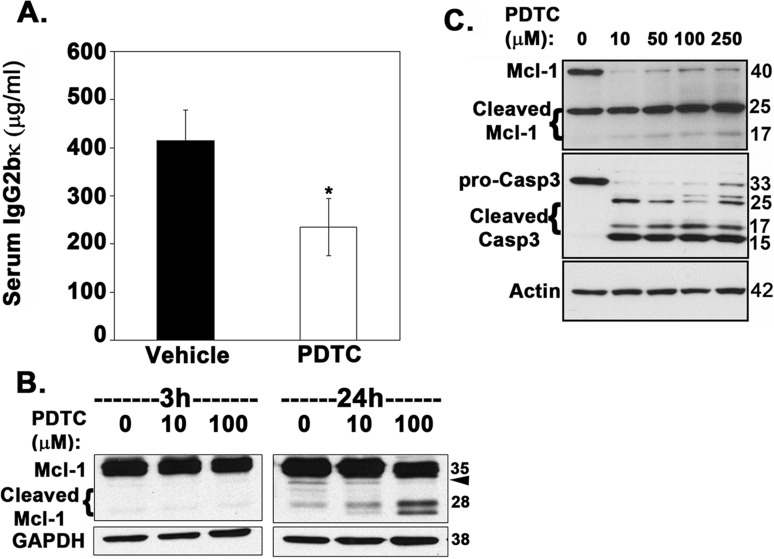
Inhibition of β-TrCP by PDTC significantly reduces overall tumor burden in myeloma-bearing mice 5TGM1-bearing mice were injected with either PDTC dissolved in saline (prepared fresh as needed and injected intraperitoneally) or PBS (*n* = 5 each). **A.** Serum IgG2bκ levels were measured 30 days after tumor cell inoculation. Data represent mean ± SEM; *, *P* < 0.05 **B.** Immunoblotting of 5TGM1 lysates shows that PDTC promotes dose-dependent cleavage of Mcl-1. Arrowhead points to Mcl-1v (32 kDa) **C.** U266 cells were treated with 10, 50, 100 and 250 μM PDTC for 24 h and lysates probed for Mcl-1 and caspase-3.

Our data consistently indicated that inhibition of β-TrCP by expression of the dominant-negative FWD1ΔF mutant decreased tumor burden in mice. Therefore, we investigated the antitumor efficacy of PDTC, a small-molecule inhibitor of β-TrCP ligase activity [50] in the 5TGM1 mouse model of myeloma. Exposing RPMI-8226 myeloma cells to PDTC results in apoptosis [51], as does treating 5TGM1 cells with PDTC (Figure S2A). Therefore, as proof of principle, we treated mice inoculated with 5TGM1 cells with PDTC. After 4 weeks, tumor burden, as assessed through serum IgG2bκ levels, was significantly lower in PDTC-treated mice than in vehicle-treated animals (Figure 6A). To determine that PDTC was not toxic to non-tumor cells in the medullary cavity at doses (10-50 μM) that kill multiple myeloma cells (Figure S2A), we performed a neonatal mouse calvarial assay. PDTC at 100 μM induced a robust increase in cell proliferation and new bone formation (Figure S2B). Immunoblotting of 5TGM1 cells treated with PDTC showed dose-dependent cleavage of Mcl-1 to proapoptotic fragments and decrease in Mcl-1v levels after 24 h of treatment (Figure 6B), consistent with the constitutively higher levels of cleaved Mcl-1 observed in 5TGM1-ΔF cells (Figure 5D). To further validate our results in human myeloma cell lines, we treated U266 cells with 10, 50, 100 and 250 μM PDTC for 24 h. Similar to 5TGM1-ΔF cells, we observed an increase in cleaved Mcl-1 and cleaved caspase-3 (Figure 6C).

## DISCUSSION

The dramatic response of multiple myeloma patients to bortezomib validates the Ub-proteasome system as a target for anticancer therapy. Recent approaches to decrease bortezomib side-effects include using a lower dose of bortezomib in conjunction with puromycin that triggers proteotoxic stress and synergistically enhances the tumoricidal effect of the proteasome inhibitor [52]. However, molecularly-targeted therapies that also reduce overall drug burden will be more clinically useful. Herein, we present evidence from proof-of-principle studies that selective inhibition of a single molecular target, SCFβ-TrCP/FWD1, upstream of the proteasome in myeloma cells mimics the anti-tumor effect of proteasome inhibition. β-TrCP1/FWD1 mediates ubiquitination and degradation of IκB, reducing total IκB levels, and we show that the dominant-negative ΔF mutant causes accumulation of total IκB. The nfkb2 gene product p100 and its N-terminal processed form p52 are critical for myeloma growth and survival [16, 17]. p100 is necessary and sufficient as an IκB protein for noncanonical NF-κB signaling [53]. In most cell types other than B-lineage cells, p100 is readily detectable, but little or no p52 is present under basal conditions [54]. By contrast, malignant plasma cells express high levels of p52 with little or no p100 present under basal conditions. Expression of the dominant-negative ΔF mutant of FWD1 in 5TGM1 myeloma cells reduced p100 processing, resulting in its accumulation. p100 is proapoptotic [55], and its accumulation in 5TGM1-ΔF cells may serve further to inhibit growth of the 5TGM1-ΔF cells as observed in our studies. β-TrCP1/FWD1 also regulates ubiquitination of ATF4 and β-catenin [12] and the data presented herein are consistent with these results.

We also examined the effect of ΔF on myeloma tumor growth and progression *in vivo* in two different models, a disseminated myeloma model with widespread tumor foci in the skeleton and a subcutaneous plasmacytoma model. In both models, as assessed by multiple parameters, myeloma tumor cell growth was almost completely inhibited in mice inoculated with 5TGM1-ΔF cells compared with 5TGM1-EV myeloma cells. Collectively, these data strongly suggest that the profound antitumor effect of the dominant-negative ΔF is mediated, in part, by tumor-secreted TNFs and is cell autonomous. Myeloma-secreted TNFs can also induce BMSCs to secrete IL-6 (the main secreted myeloma cell growth and survival factor) locally and modulate cell-cell adhesion between myeloma cells and BMSCs in the bone microenvironment [21]. However, because ΔF's effect is just as profound in a setting devoid of BMSCs, and because our studies did not indicate any major effects of IL-6, the potential mechanism of action of TNF in this context is quite likely independent of its known paracrine effects.

Because we observed increased sensitivity of 5TGM1-ΔF cells to apoptosis, we examined reported β-TrCP targets that have been implicated in apoptosis. We found that levels of the anti-apoptotic proteins, Bcl-2 and cIAP, were reduced compared to empty vector controls, and conversely, we observed increased stability of procaspase-3 as well as its increased cleavage in 5TGM1-ΔF cells. Total expression of the anti-apoptotic protein, Mcl-1, increased significantly (Figure 5A), perhaps due to increased ATF4 accumulation (Figure 1D) and its consequent binding to Mcl-1 regulatory site [56] and translational activation. However, 5TGM1-ΔF cells not only show a significant decrease in the anti-apoptotic Mcl-v isoform but also exhibit an increase in cleaved, pro-apoptotic Mcl-1.

Taken together, our results strongly indicate that perturbation of β-TRCP/FWD1 ligase activity suffices to trigger apoptotic myeloma cell death *in vivo* in a cell-autonomous manner, emphasizing the critical role of the tumor microenvironment in the fate of myeloma cells. Importantly, we also demonstrate an anti-tumor efficacy of PDTC, a small-molecule that has been reported to inhibit β-TrCP E3 ubiquitin ligase activity [50] in the well-established preclinical 5TGM1 mouse model of multiple myeloma that has been shown in the past to be predictive of drugs with clinical utility. Interestingly, PDTC is an analog of disulfiram, a drug used in the treatment of alcoholism that that been also shown to be toxic to human and murine myeloma cells *in vitro* [51, 57]. There is increasing interest in the development of anti-cancer therapies that target SCF Ub ligases [58-60]. Our data herein clearly identify β-TrCP/FWD1 as one such selective target in the Ub-proteasome pathway that could be exploited for therapeutic benefit in MM and perhaps other hematologic malignancies.

## MATERIALS AND METHODS

### Ethics statement

This study was carried out in strict accordance with the recommendations in the Guide for the Care and Use of Laboratory Animals of the National Institutes of Health. The protocols described below were approved by the Institutional Animal Care and Use Committee (IACUC) at The University of Texas Health Science Center at San Antonio (UTHSCSA). The Laboratory Animal Resource facilities at UTHSCSA are accredited by the Association for Assessment and Accreditation of Laboratory Animal Care International (AAALAC). All efforts were made to minimize suffering.

### Cell culture

The 5TGM1-GFP cell line, a genetically modified subclone of 5TGM1 mouse myeloma cells from the Radl 5T33 (IgG2b-secreting) myeloma previously established in our laboratory [24, 26, 61-63], stably expresses enhanced green fluorescent protein (GFP) [26] and is maintained in RPMI 1640 medium containing 10 mM HEPES (American Type Culture Collection, Manassas, VA) and 15% fetal calf serum (Summit Biotechnology, Fort Collins, CO). To generate 5TGM1-GFP cells stably expressing FWD1ΔF, 5 × 10^5^ cells per 60-mm cell culture dish were plated and left overnight. Flag-tagged pcDNA3 FWD1ΔF or empty vector were then transfected into the cells using DMRIE-C reagent (Life Technologies, Grand Island, NY) and selection was performed using geneticin (G418; 500 μg/ml) to obtain 5TGM1-ΔF or 5TGM1-EV cell clones, respectively. Stable gene expression was verified by immunoblotting with an anti-Flag M2 antibody (Sigma, St. Louis, MO), as described previously [12]. U266 human myeloma cells (obtained from ATCC) were grown in RPMI 1640 medium containing 15% fetal calf serum. To determine the effect of pyrrolidone dithiocarbamate (PDTC; Sigma Chemicals, St. Louis, MO), cells were plated at 0.4 × 10^6^ cells in T-25 flasks and treated with the indicated doses of PDTC (dissolved in DMSO) for 24 h. Cells were harvested by centrifugation, lysed with RIPA buffer, sonicated and supernatant used for immunoblotting

### Immunoblotting

Whole-cell lysates prepared in radioimmunoprecipitation assay (RIPA) lysis buffer containing protease inhibitors (Roche, Basel, Switzerland) were resolved on 10% or 13% polyacrylamide gels and transferred to a polyvinylidene difluoride membrane. Membranes were blocked with 5% milk in Tris-buffered saline containing 1% Tween 20 for 1 h, incubated with primary antibodies in blocking solution overnight, and washed and incubated with appropriate secondary antibodies at room temperature for 1 h. An enhanced chemiluminescence system (Perkin-Elmer, Waltham, MA) was used to visualize signals. For normalization, membranes were stripped and reprobed for actin or glyceraldehyde-3-phosphate dehydrogenase (GAPDH). Antibodies against NF-κB1 p50, NF-κB2 p52, Bcl-2, GAPDH, ATF4, β-catenin and actin were from Santa Cruz Biotechnology (Santa Cruz, CA); antibodies against caspase-3, phosphorylated β-catenin, IκBα and XIAP were from Cell Signaling Technology (Danvers, MA). Antibody to Mcl-1 was from Rockland Immunochemicals (Gilbertsville, PA). Antibody against pan-cIAP was from R&D Systems (Minnesota, MN).

### Cell viability assay

Cell viability was assessed using a CellTiter 96 Aqueous Nonradioactive Cell Proliferation Assay (Promega, Madison, WI). In brief, cells cultured in 100 μl of media in 96-well plates were pulsed with 10 μl of tetrazolium salt (MTS) for the final 4 h of the incubation period at 37°C and cell viability determined by measuring absorbance at 490 nm on a microplate reader (BioTek Instruments, Winooski, VT). In parallel, trypan blue-stained cells were counted using a Neubauer hemocytometer to confirm loss of viability. All experiments were repeated at least three times, with each condition tested in quadruplicate.

### Apoptosis and cell cycle analyses

To quantify apoptosis, cells were washed and resuspended in ice-cold annexin V binding buffer containing phycoerythrin-conjugated annexin V and 7-amino-actinomycin D (7-AAD; BD Pharmingen, San Jose, CA) and analyzed them immediately by dual-color flow cytometry on a FACS Calibur (Becton Dickinson, San Jose, CA). Cells gated exclusively as annexin V^+^, 7-AAD^−^ were classified as early apoptotic and those gated as annexin V^−^, 7-AAD^+^ as necrotic. Apoptosis was also independently confirmed using standard morphological criteria, including nuclear chromatin condensation and presence of apoptotic bodies in cells cytospun onto glass slides and stained with hematoxylin and eosin (H&E). For cell cycle analysis, treated cells were washed in phosphate-buffered saline (PBS), fixed in ethanol, pelleted and labeled with 50 μg/ml of propidium iodide in the presence of 1 mg/ml of RNase A for 30 min in the dark at room temperature, followed by gating for single cells.

### Coculture experiments

GFP-expressing 5TGM1-EV or 5TGM1-ΔF (5 × 10^4^ cells/well) were seeded onto monolayer of 14M1 bone marrow stromal cells (BMSCs) at approximately 60% confluence in six-well plates and treated with recombinant murine tumor necrosis factor α (TNF-α; 20 ng/ml; R&D Systems). 14M1 BMSCs were originally isolated from C57Bl/KaLwRij mice bearing 5T myeloma [24, 64, 65]. After 72 h, spent media were replaced with fresh media (to remove floating/dead cells) and a Zeiss Axiovert 25 CFL inverted microscope (Carl Zeiss Microscopy, Thornwood, NY) was used to image green fluorescent cells.

### Animal studies

Experiments were performed with weight-matched, 6-10 weeks old female syngeneic C57BL/KaLwRij mice (Harlan, The Netherlands).

#### Disseminated myeloma bone disease model

10^6^ 5TGM1-EV or 5TGM1-ΔF cells in 200 μl of PBS were inoculated into mice through tail veins. Whole-blood samples were collected for sera by retro-orbital puncture under methoxyflurane-inhaled anesthesia at baseline and then weekly after tumor inoculation. Serum monoclonal paraprotein (IgG2bκ) titers were measured using an “in-house’ specific two-site enzyme-linked immunosorbent assay with rat anti-mouse IgG2b (Zymed Labs, San Francisco, CA) and HRP-conjugated rat anti-mouse IgG (BioDesign, Kennebunk, ME) as capture and detection antibodies, respectively. Experiments were terminated after 30 days. Because tumor-bearing mice in this myeloma model also develop splenomegaly, spleens were also weighed immediately after sacrifice.

To determine whether pyrrolidine dithiocarbamate (PDTC; Sigma) could inhibit tumor growth, 5TGM1 cells were inoculated into C57BL/KaLwRij mice through tail veins. Mice were then randomly assigned to one of two groups (*n* = 5 each) and treated with either PDTC (50 mg/kg of body weight) or vehicle (PBS) daily 5 days per week for 4 weeks by intraperitoneal injection. IgG2bκ titers were measured in sera obtained 30 days after tumor cell inoculation.

#### Subcutaneous plasmacytoma model

Plasmacytomas were induced by injecting 5TGM1-GFP cells in 100 μl of PBS subcutaneously over flanks of naïve C57BL/KaLwRij mice. Tumor diameters were taken in three dimensions on days 14 and 21 post-tumor cell inoculation using an electronic caliper. Measurements of the longest perpendicular tumor diameters were used to estimate tumor volumes using the formula for volume of an ellipsoid: 4/3π (width/2)^2^ × (length/2).

#### Histology and histomorphometric analysis

Skeletal tissues were fixed in 10% buffered formalin, decalcified in 14% EDTA, embedded in paraffin, and sectioned at 4 μm along the midsagittal plane. Sections were stained with hematoxylin and eosin (H&E) and analyzed for tumor infiltration of bones stereologically using an Olympus BX40 microscope (Olympus, Center Valley, PA, USA) connected to desktop computer running the OsteoMeasure histomorphometry software (Osteometrics, Atlanta, GA, USA) [24, 26, 61]. Consecutive sections of tibiae and femora were stained for tartrate-resistant acid phosphatase (TRAP) and TRAP+ multinucleated cells (nuclei ≥ 3) counted as osteoclasts. Soft tissues were also embedded in paraffin and tumor volume assessed as described above. In all cases, at least three sections from non-consecutive levels were quantified and tumor volume calculated as tumor area/total bone marrow cavity area is expressed as mean ± SEM.

### Statistical analysis

Unless otherwise stated, data were analyzed using a one-way analysis of variance with Fisher's post-hoc least-significant difference test and values of *P* ≤ 0.05 accepted as significantly different.

## SUPPLEMENTARY MATERIAL FIGURES


