# Supplementary material for: A dominant-negative F-box deleted mutant of E3 ubiquitin ligase, β-TrCP1/FWD1, markedly reduces myeloma cell growth and survival in mice
Source: Oncotarget. 2015 May 12;6(25):21589–602. doi: 10.18632/oncotarget.4120 (PMC4673288; doi:10.18632/oncotarget.4120)
Supplement: Supplementary file 1 [file oncotarget-06-21589-s001.pdf]

A dominant-negative F-box deleted mutant of E3 ubiquitin ligase, β-TrCP1/FWD1, markedly reduces myeloma cell growth and survival in mice

Supplementary Material

RESULTS

All 5TGM1-EV and 5TGM1-ΔF cells express GFP

Fig. 4C shows that TNF-α significantly decreases GFP-expressing 5TGM1-ΔF cells. To confirm that GFP can be used as a marker for cell viability, we simultaneously performed phase-contrast and fluorescence microscopy. As shown in Figure S1, panels a,c,e and g are phase-contrast photomicrographs of panels b, d, f and h respectively that demonstrate that there were little or no viable tumor cells that did not express GFP in any of the conditions used in our assay. Therefore, we could use GFP expression as an adequate measure to determine cell viability.

PDTC is not toxic to non-tumor cells at doses lethal to multiple myeloma cells

To determine if the toxic effects of PDTC on multiple myeloma cells was systemic and affected non-tumor cells, we quantified 5TGM1 cell viability at different doses of PDTC using the MTT assay. As shown in Figure S2A, 50 µM PDTC killed approximately 70% of 5TGM1 cells. Next, we determined if PDTC was toxic in vivo using a neonatal mouse calvarial assay, a well-established method [1-3]. Interestingly, even a 2-fold increase of that dose was not only non-toxic to other types but also stimulated robust cell proliferation and new bone formation (Figure S2B). This is consistent with our previous published data showing that the proteasomal inhibitor, bortezomib, at a concentration that kills tumor cells has a similar bone anabolic effect [4]. Hence, it is unlikely that general toxicity in the medullary cavity is one of PDTC's mechanism of action.

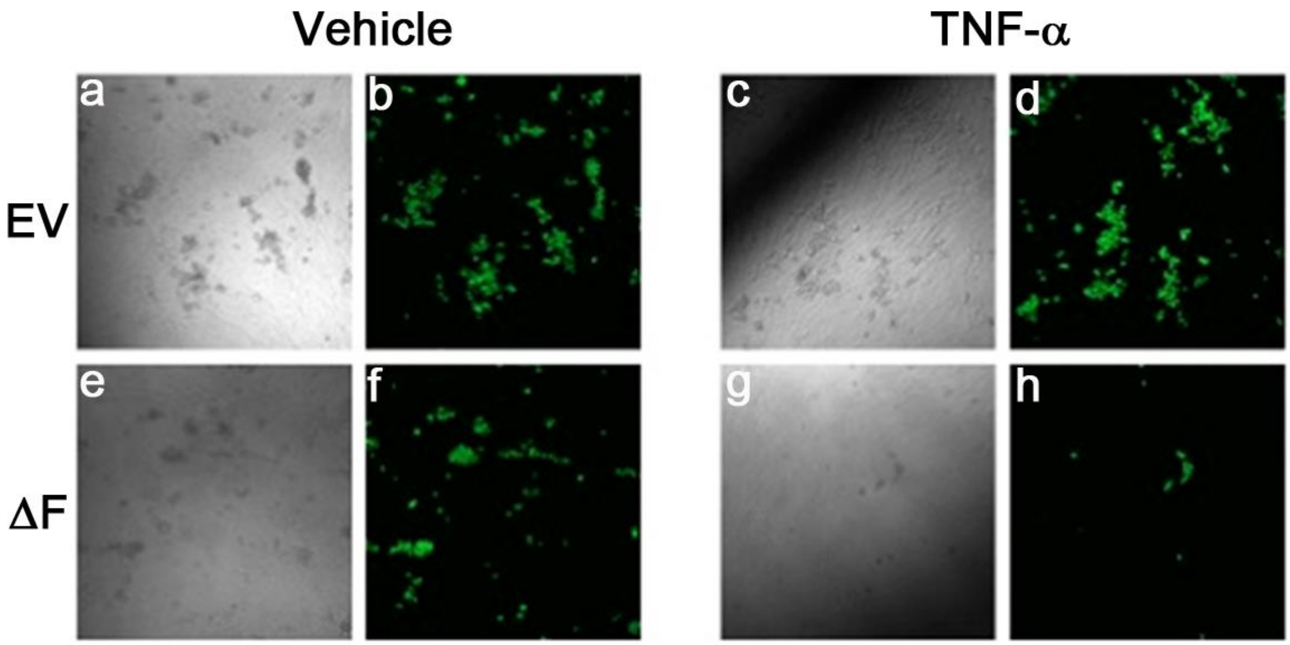

S1: All 5TGM1-EV and 5TGM1-ΔF cells express GFP. Panels a,c,e and g are phase-contrast photomicrographs of the fluorescent images shown in panels b, d, f and h respectively.

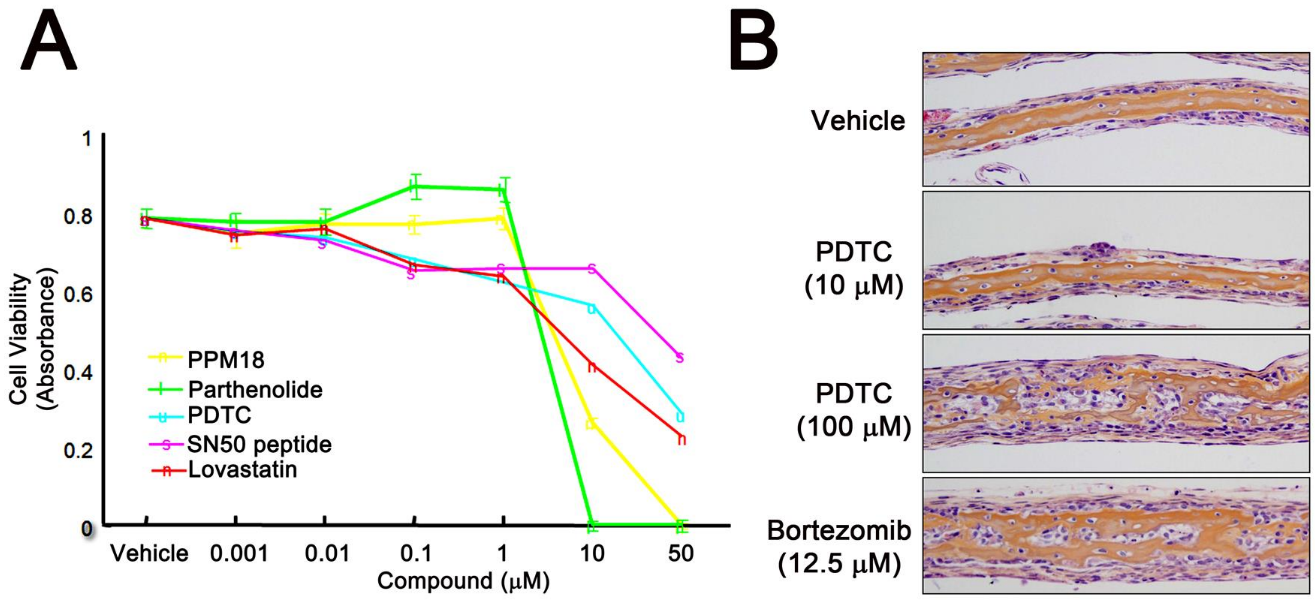

S2: PDTC is not toxic to non-tumor cells at doses lethal to multiple myeloma cells. A. Cells were cultured in 96-well plates and treated with the indicated doses of PDTC and other chemicals for 24 h. For the final 4 h of the incubation, cells were pulsed once with 10 µl of tetrazolium salt (MTS) and cell viability determined by measuring absorbance at 490 nm. Data, shown as mean ± SEM, is an average of three experiments with each condition tested in quadruplicate. **(B)** H&E stained sections of neonatal mouse calvariae after treatment with vehicle, PDTC or bortezomib at the indicated concentrations for 72 h. Note the PDTC-mediated dose-dependent increase in cell numbers and new bone formed indicating that PDTC is not toxic to non-tumor cells.

MATERIALS AND METHODS

Neonatal mouse calvarial bone assay

As described previously [1-3], neonatal mouse calvariae from 4-day old Swiss pups (Institute for Cancer Research; ICR) were excised and hemi-calvariae explanted on metal grids that overlay BGJ medium with Fitton-Jackson modification (Sigma) containing 0.1% BSA and glutamine. Calvariae were incubated at $37^{o}C/5\% \; CO_{2.}$ After 24 h, calvariae were transferred to wells with medium containing the indicated test compound or vehicle. After 72 h exposure, calvariae were fixed in 10% buffered formalin for 24 h, decalcified in 14% EDTA overnight, and embedded in paraffin. Sections (7 μm) were cut and stained with hematoxylin and eosin (H&E).

REFERENCES

1. Garrett IR, Chen D, Gutierrez G, Zhao M, Escobedo A, Rossini G, Harris SE, Gallwitz W, Kim KB, Hu S, Crews CM and Mundy GR. Selective inhibitors of the osteoblast proteasome stimulate bone formation in vivo and in vitro. J Clin Invest. 2003; 111(11):1771-1782.

2. Mohammad KS, Chirgwin JM and Guise TA. Assessing new bone formation in neonatal calvarial organ cultures. Methods Mol Biol. 2008; 455:37-50.

3. Mundy G, Garrett R, Harris S, Chan J, Chen D, Rossini G, Boyce B, Zhao M and Gutierrez G. Stimulation of bone formation in vitro and in rodents by statins. Science. 1999; 286(5446):1946-1949.

4. Oyajobi BO, Garrett IR, Gupta A, Flores A, Esparza J, Munoz S, Zhao M and Mundy GR. Stimulation of new bone formation by the proteasome inhibitor, bortezomib: implications for myeloma bone disease. Br J Haematol. 2007; 139(3):434-438.
